# Treatment with Mycophenolat Mofetil of Steroid-Dependent Asthma—One Case of Severe Asthma

**DOI:** 10.1155/2009/821013

**Published:** 2009-10-07

**Authors:** V. Backer, E. Hjardem, T. Karlsmark

**Affiliations:** ^1^Department of Respiratory Medicine, Copenhagen University Hospital, Bispebjerg Hospital, DK-2400 Copenhagen, Denmark; ^2^Department of Dermatology, Copenhagen University Hospital, Bispebjerg Hospital, DK-2400 Copenhagen, Denmark

## Abstract

*Background*. Some patients with severe nonallergic asthma can be difficult to treat with conventional therapy. Mycophenolat Mofetil (MMF) is an immunosuppressive drug with multiple mechanisms. There is theoretical support of specific effect of MMF on severe asthma, in “difficult to treat” patients. The aim of the present case was to explore whether MMF had an effect in one case of severe refractory asthma. *The patient*. This case deals with one patient with very severe nonallergic treatment refractory asthma who experienced treatment failure on ordinary antiasthmatic treatment and severe adverse events to conventional immunosupressive treatment. She was then treated with MMF. *Results*. The patient experienced a gain in FEV1 and a reduction in the need for oral glucocorticosteroids as well as seldom need of when needed bronchodilator both during daytime and night. It therefore seems very interesting to examine the use of MMF for severe refractory asthma with further clinical studies and basic cellular trials.

## 1. Introduction

Asthma is an inflammatory disease characterised by bronchial hyperresponsiveness and airway obstruction. Five to ten percent of asthma patients have in spite of relevant treatment severe asthma with chronic inflammation causing airway remodelling and persistent loss of lung function [1].

The GINA guidelines recommended a stepwise treatment approach, which at step 4 includes short-acting beta-agonist (SABA), inhaled glucocorticosteroids (ICS) in combination with long-acting inhaled beta2-agonist (LABA), as well as oral theophyllin [2]. However, in severe cases with poor controlled asthma, although maximally treated, additional treatment with oral glucocorticosteroids (OCS) is needed [3], even though side effects of long-term treatment are health damaging. Although, the GINA guidelines try to be covering the entire field, some suggest that it might be to superficial and not cover all possibilities [4].

Thus different types of immunosuppressive drugs have been tested [5, 6] in order to gain disease control severe persistent asthma. The commonly used Immunomodulatory therapy is primary Azathioprine (ATZ) or methotraxate (MTX) [4]; however in dermatology they have shifted from these drugs to other therapeutic possibilities with a more acceptable profile with fewer side effects. One of these are Mycophenolat Mofetil (MMF), and this drug could also be a possible drug of choice in asthma treatment as MMF has several immunosuppressant actions in animal models and in “in vitro” trials: it inhibits the proliferation of T- and B-lymphocytes, increases apoptosis in T-lymphocytes, inhibits initiation of and suppresses ongoing immunoglobulin production, interferes with the maturation process of dendritic cells, inhibits the adhesion and penetration of monocytes and T-lymphocytes by the inflammatory site, and inhibits the inducible Nitric Oxide (iNOs). It is believed that T-lymphocytes play an important role in asthma and that T-lymphocytes resistant to corticosteroids can be suppressed by other immunosuppressive agents, for example, by MMF [7, 8]. Furthermore, MMF is also expected to decrease the production of the inflammatory promoters TNF alpha and Interleukin 1 [9, 10]. Besides the immunosuppressive mechanisms, MMF seems to have an antiproliferative effect, for example, on the smooth muscle cells, endothelial cells, and fibroblasts [10], which could help preventing the airway remodelling of severe asthma.

So far MMF has mostly been used for prevention of acute and chronic allograft rejection, but indications such as diabetic nephropathy (animal trials), lupus nephritis (human trials), rheumatoid arthritis (human trials), ocular inflammation (human trials), myasthenia gravis (human trials), several skin diseases (human trials), and cytomegalovirus infections (human trials) have been suggested [9–11].

Lastly, two studies have shown that MMF is able to suppress inflammatory activity in leukocyte cultures from asthma patients [7, 8] and seems to be relatively safe, the main adverse events being gastrointestinal inconvenience and mild reversible bone-marrow suppression [8–11]. However, little is known about the long-term effects and side effects. 

## 2. The Case

A 54-year-old woman, known with asthma for ten years, was referred to a Department of Respiratory Medicine because of severe asthma, which was difficult to treat. She had a smoking history of five packyears but had stopped ten years before. She had no allergies, had two family members with severe asthma, and worked as an office assistant with no work-related toxic exposure. Her asthma had been difficult to control in spite of relevant treatment, she had had five oral courses of glucocorticosteroid within the last six months prior to referral to the University hospital, and she had been on a part-time sick leave for approximately six months prior to referral. She had gained in weight over a ten-year period from 59 kg to 110 kg.

### 2.1. Prior to Referral

Her FEV_1_ had varied between 1.4 L (53%) and 1.7 L (64%) in the last six months at her GP. Her daily treatment included ICS (budesonid 640 *μ*g), LABA, short acting anticholinergica, and SABA 8–12 times daily, when needed.

### 2.2. At Referral

Basic evaluations were performed, such as Chest X-ray, Electrocardiogram, Echocardiography, alpha1-antitrypsine, and Allergy tests which all were normal. The Computer Tomography (CT) scan showed discreet lung emphysema, indicating hyperinflation. 

Her lung function was measured, and FEV_1_ increased from 1.35 L (50%) to 1.85 (70%) after four puffs of beta_2_-agonist; the post bronchodilator FVC was 2.57 (81%) and FEV_1_/FVC ratio was 92%. Initially, her ICS dose was changed to mometason furoat (800 *μ*g) combined with LABA twice daily and theophyllin (300 mg) as well as long-acting muscarine antagonist (LAMA) was added.

After two months of follow-up at the out-patient clinic her lung function had further deteriorated as FEV_1_ was 1.22 (46%) and one month later as low as 0.96 L (36%). She had many daily asthma attacks as well as night time symptoms. The night time awakenings due to asthma happened almost every night. She was given a nebuliser to treat acute attacks. Due to the further deterioration in lung function, her treatment with ICS was switch back to budesonide as well as increased in dosis and oral corticosteroid was added.

After four months of unsuccessful asthma treatment her asthma continued to be uncontrolled. Azathioprine (100 mg) was added but after two months of treatment it was withdrawn again due to intolerable adverse events (nausea, vomiting, diarrhoea) and furthermore, lack of effect on the asthmatic disease. The OCS dose was during this period fluctuating between 25 mg and 12.5 mg daily.

After six months of follow-up, on high doses of ICS, continued doses of OCS, theophyllin, LAMA, LABA, SABA, and nebulised SABA when needed—still without having controlled her asthma—we decided to start her on another immunosuppressive treatment and we wanted to explore the effect of MMF treatment.

Prior to MMF treatment the patient had a fiberoptic bronchoscopia performed with mucosa biopsies and bronchial lavage, she had her eNO and lung function measured, and blood samples taken including blood cell count, eosinophilic chemotacktic protein (ECP), and alpha-1-antitrypsine, all of them were close to normal. On oral corticosteroid treatment, the bronchial mucosal biopsies were normal, apart from mild oedemas, and the lavage fluid showed few neutrophiles but no eosinophiles. Blood leukocytes were slightly raised. All other blood samples were normal, and eNO was not increased.

### 2.3. Mycophenolat Mofetil

A dose of 500 mg MMF was initiated. Within the first week the patient's FEV_1_ rose from 1.38 (52%) to 2.03 (76%) in spite of a decreased OCS dose to 15 mg daily. Her peak flow increased from 284 (73%) to 417 (107%), her symptoms diminished, her night time symptoms disappeared, and she had less need of SABA. MMF was further increased to 1000 mg bid, and OCS was kept on 15 mg daily.

Since the beginning of MMF treatment she has experienced better asthma control. She has during the last year had one exacerbation with pulmonary infection, high fever, and deterioration in lung function, after which her MMF doses were reduced to 750 mg bid. But in general, her asthma is now well controlled, with few asthma symptoms and hardly any use of extra SABA. She still uses moderate doses of ICS, LABA, and LAMA. Her FEV_1_ has been unchanged (60%–70%), PEF has stabilised on 400 L/sec, very few extra puffs of SABA are needed, and her systemic steroid has been kept on 5 mg, and MMF on 750 mg bid. Since the start of MMF and the reduction in OCS, she has lost 20 kg in weight and have takenup her work at 50% or more. She is having few, if any, seek leave days from work due to asthma and she have started physical rehabilitation.

## 3. Discussion

This case shows an example of severe nonatopic asthma, which was difficult to control with conventional therapy due to treatment failure and adverse events.

Drugs such as Tacrolismus, Azathioprin, Methrotraxat, Cyclosporine, and MMF are all immunosuppressive agents that have been suggested for the treatment of severe refractory asthma, but none of them have been tested for this purpose. We decided to explore the effect of MMF because it—at least in theory—seems reasonable as an antiasthmatic treatment. Furthermore, a substantial shift to MMF in dermatology, when treating severe uncontrolled atopic eczema in adults, has occurred.

Uncontrolled asthma could besides being difficult to treat, be caused by low adherence to the antiasthmatic treatment. Adherence is a big issue in asthma treatment, as it is well known at least in the adolescents, and the young adults are having an adherence rate of 40%–70%. However the present patient had gain almost 60 kg in weight during a period of 10 years and repetitive steroid courses. Furthermore, she has deterioration in lung function and was threaded of losing her job, and her family was anxious of her severe exacerbations twice a month. Although we never in clinical practise in adult outpatients clinics count the use of inhaled steroid puffs, we do believe that she was rather adherent.

In this patient, we saw an immediate effect of MMF with a decrease in subjective symptoms and SABA requirement as well as a substantial rise in FEV_1_. The basic pathology in asthma behind this immediate effect is unknown, but knowledge from the use of MMF in preventing graft rejection supports a quick onset of action of the drug [9] but it is difficult to predict exactly which of the anti-inflammatory mechanisms is responsible for this. We cannot explain this immediate effect, and although the mechanism behind this is unknown, it has been a stable improvement with fewer and minor exacerbation than prior to MMF. Furthermore, on a longer term, the drug was able to stabilize the patients' asthma on a substantially reduced dose of OCS, followed by a weight reduction of 20 kg, with only one exacerbation during the year of follow-up, and there have been no detectable adverse events.

Since this observation is based on only one case study, it is possible that the improvement after initiating MMF is due to an effect of frequent visit and personal caretaking or a “regression towards the mean.” It is also possible that an increase in compliance could have played a part in the variations of her lung function and symptoms, although this patient was followed closely during the entire followup period, had a good inhalation technique, and was motivated to follow the different treatment changes from the start. Furthermore, Azathioprine did not show the same effect but caused adverse events in this patient. On the other hand, during the entire period of MMF treatment there had been no gastrointestinal side effects, and furthermore no signs of bone-marrow depression.

When the MMF treatment was initiated in this patient, she did not have an increased level of exhaled eNO. This can be explained by her constant OCS treatment, which probably partly suppressed the inflammation but could also be that this patient had a neutrophilic asthma which probably would show a lower level of eNO.

From this case, no predictions regarding the long-term effects or side effects of MMF, the glucocorticosteroid-sparing ability, can be made. Nevertheless, since the theory supports specific effect of MMF, this case indicates an effect of the drug, and due to a great need for alternative treatments, it seems interesting to examine the use of MMF for severe refractory asthma further clinical studies and basic cellular trials.
